# Tetanus-cure by a Sudden Shock to the Nerves

**Published:** 1896-06

**Authors:** 


TETANUS-CURE BY A SUDDEN SHOCK TO THE NERVES. The report of an original cure for tetanus by a sudden and very severe shock to the nerve-system, in No. 21 (1895) of the *Thierärzt Centralblattz*, recalls to my mind a similar occurrence which was related to me while a student by the deceased veterinary surgeon, P. Johnson Zimmer, who lived in my native village, Pechtoldsdorf, in the neighborhood of Vienna. The dead colleague, whose honorable character is well-known to many members of the profession still living, related to me that he cured a horse which had already become stiff by exciting him highly by firing off a pistol several times quickly in succession, in the stall beside him. This caused a severe sweat to break out upon the horse, and the cure consisted in pouring cold water over the horse from a considerable height and then rubbing him down. The result was that the horse recovered. Where Zimmer learned this cure I have forgotten. However senseless this treatment of tetanus appears in regard to the apparently contradictory effect in the use of powerful means of excitement, yet the occurrence of reports to the same effect from different places is at least worthy of consideration, and I would not hesitate to try it with the full knowledge of the owner.—*Ibid.*

